# Journal/Author Name Estimator (JANE)

**DOI:** 10.5195/jmla.2019.598

**Published:** 2019-01-01

**Authors:** Carolann Lee Curry

**Affiliations:** Library Assistant Professor and Reference, Outreach, and Assessment Librarian, Skelton Medical Library, Mercer University School of Medicine, Macon, GA, curry_cl@mercer.edu

## OVERVIEW

The Journal/Author Name Estimator (JANE) is a free online bibliographic journal selection tool. Journal selection tools, also known as journal matching or journal comparison tools, are popular resources that help authors determine the most appropriate in scope journal to publish their manuscripts. JANE is one of the earliest journal selection tools, debuting in 2007 [1]. The resource is web-based and allows users to input keywords, abstract text, or author names and view related articles based on user-supplied terms. At the time of this writing, no formal mobile app or browser extension has been developed to utilize the resource. There is an application programming interface (API) freely available in beta version that is available to users who want to integrate JANE into their own applications.

## CONTENT

JANE interfaces directly with the PubMed operating from the PubMed/MEDLINE data set, meaning both MEDLINE-indexed journals as well as articles deposited into PubMed Central can be retrieved when searching the resource. JANE’s indexing criteria include journals from PubMed/MEDLINE that contain abstracts published within the past ten years. JANE does not search categories that are not viewed as original research. For example, editorials, newspaper articles, comments, conferences, directories, retractions, errata, and so on are omitted. During a search, JANE uses the Lucene open source search engine to search for the most similar fifty articles based on user input and assigns similarity and confidence scores, which determine the search result order.

Besides journal comparison functions, other uses of the JANE resource include convenient identification of related articles that authors can read and/or cite in their manuscripts, as well as aggregation of authors who could potentially serve on journal review boards. For example, publishers who need to fill editorial or peer-reviewer positions can search JANE, using the author search to identify relevant subject specialists [2].

## FEATURES

**Figure 1:**
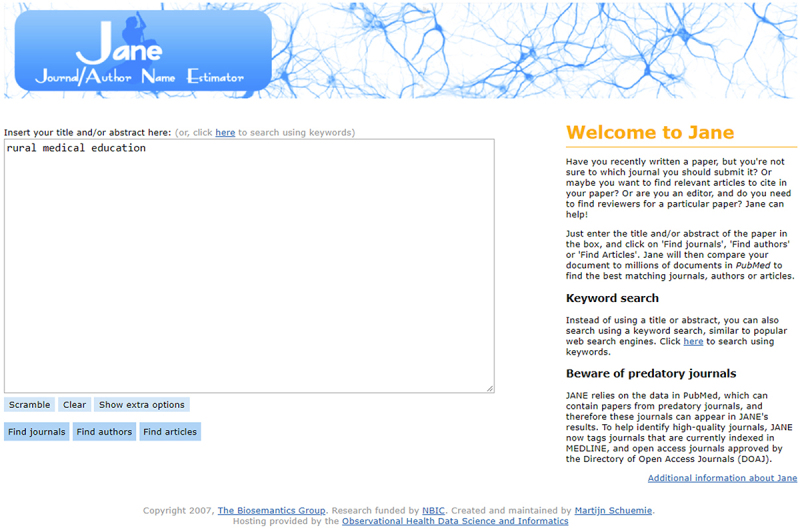
Journal/Author Name Estimator (JANE) interface

JANE’s simple search interface allows users to easily input data into an open text box. The home page search field defaults to a larger expanded “Title and/or Abstract” search box. Users can also select the keyword link to be taken to a smaller text box where keyword terms can be searched. Keyword searching also works when text is entered in the “Title and/or Abstract” search box (Figure 1).

Both search boxes include a “Show extra options” button, where users can limit results by language (English, French, German, Italian, Japanese, Russian, and Spanish); publication type (case reports, various phases of clinical trials, meta-analyses, reviews, etc.); open access journal options; and journals only indexed for PubMed Central.

**Figure 2:**
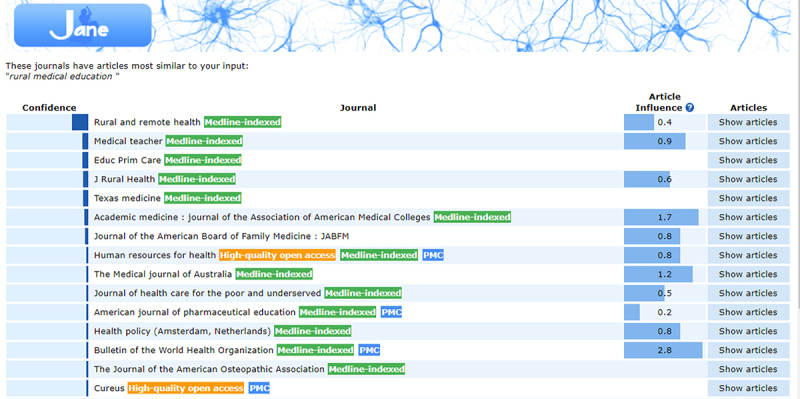
JANE “Show articles” link

Each search box also includes options to either “Find journals,” “Find authors,” or “Find articles” depending on the query users want to search. “Find journals” retrieves a list of journals that are most similar to the user’s input terms. Journals are sorted by confidence, with Eigenfactor article influence (AI) metrics displayed when available. A “Show articles” link is also displayed, which retrieves a list of relevant articles from each journal, listed by confidence (Figure 2). Users can also select individual articles from the results list, which opens a new browser tab taking users to the full record in PubMed.

The “Find authors” link displays a list of published authors based on the input data. There is an email option to directly contact each author. The “Show articles” option works similarly to the “Find journals” option and retrieves a list of articles published by the relevant authors, based on input data. Again, articles link directly out to PubMed in a separate browser tab. The “Find articles” option retrieves articles that are most similar to input.

## PRIVACY

For authors who are concerned about the confidentiality of their work, JANE does not store data sent to the resource server. As an additional measure of security, the resource offers a “scramble” feature that, when selected, alphabetizes input terms in the browser before the data are sent to the server. Schuemie notes in the FAQ section of the site, “that putting the words in alphabetical order does not completely disguise your input, but it does make it extremely hard to read, and it has no effect on the performance of JANE.” [3].

## UPDATES

While there have been minimal changes to the functionality and display of the website, in 2017, JANE began cautioning users about potential predatory publishers. In a statement appearing on the home page, JANE notes that it relies on PubMed data, acknowledging that predatory journals can appear in PubMed and, therefore, can potentially appear in JANE search results. In an effort to distinguish reputable journals from questionable ones, JANE now displays color-coded identifiers behind each journal name in the results page. The green “Medline-indexed” tab is defined as a journal currently indexed for MEDLINE. The orange “High-quality open access” tab is defined as a journal that does not charge readers or institutions for access and is considered to be of high quality according to the Directory of Open Access Journals (DOAJ). The blue “PMC” tab is defined as a journal where some or all of its articles are deposited, sometimes after a delay, in PubMed Central (PMC).

## CONCLUSION

The JANE website is no frills but achieves its goal of journal matching, journal comparison, and relevant author retrieval. While the site offers a satisfactory amount of transparency on how the resource works, users who have questions or seek clarification can reach out directly to resource creator, Martijn Schuemie, who also maintains the JANE website. An email sent during the preparation of this review was responded to within twenty-four hours. A 2011 JANE review author also noted a one-day turnaround time in responding to that reviewer’s email [4].

For those working in health and biomedical fields, JANE is a practical and streamlined journal selection tool that aggregates data directly from the PubMed/MEDLINE database. Because of this connection, results are in scope and generally more relevant when compared to other journal selection tools. As with any journal matching resource, authors should always vet journal information externally on the publishers’ pages to ensure correctness and completeness. JANE is recommended for researchers or authors looking to determine the best fit for their manuscripts as well as health sciences librarians who are working with junior researchers and others new to scholarly publishing.
